# Bacterial community and cyanotoxin gene distribution of the Winam Gulf, Lake Victoria, Kenya

**DOI:** 10.1111/1758-2229.13297

**Published:** 2024-06-17

**Authors:** Katelyn M. Brown, Katelyn B. Barker, Ryan S. Wagner, Christopher S. Ward, Lewis Sitoki, James Njiru, Reuben Omondi, James Achiya, Albert Getabu, R. Michael McKay, George S. Bullerjahn, the NSF‐IRES Lake Victoria Research Consortium

**Affiliations:** ^1^ Biological Sciences Bowling Green State University Bowling Green Ohio USA; ^2^ Great Lakes Centers for Fresh Waters and Human Health Bowling Green Ohio USA; ^3^ Department of Earth, Environmental Science and Technology Technical University of Kenya Nairobi Kenya; ^4^ Kenya Marine and Fisheries Research Institute Kisumu Kenya; ^5^ Department of Fisheries and Limnology Kisii University Kisii Kenya; ^6^ Great Lakes Institute for Environmental Research University of Windsor Windsor Ontario Canada; ^7^ NSF‐IRES Lake Victoria Research Consortium (www.agl‐acare.org/bgsu‐2022) Bowling Green State University Bowling Green Ohio USA

## Abstract

The Winam Gulf (Kenya) is frequently impaired by cyanobacterial harmful algal blooms (cHABs) due to inadequate wastewater treatment and excess agricultural nutrient input. While phytoplankton in Lake Victoria have been characterized using morphological criteria, our aim is to identify potential toxin‐producing cyanobacteria using molecular approaches. The Gulf was sampled over two successive summer seasons, and 16S and 18S ribosomal RNA gene sequencing was performed. Additionally, key genes involved in production of cyanotoxins were examined by quantitative PCR. Bacterial communities were spatially variable, forming distinct clusters in line with regions of the Gulf. Taxa associated with diazotrophy were dominant near Homa Bay. On the eastern side, samples exhibited elevated *cyrA* abundances, indicating genetic capability of cylindrospermopsin synthesis. Indeed, near the Nyando River mouth in 2022, *cyrA* exceeded 10 million copies L^−1^ where there were more than 6000 *Cylindrospermopsis* spp. cells mL^−1^. In contrast, the southwestern region had elevated *mcyE* gene (microcystin synthesis) detections near Homa Bay where *Microcystis* and *Dolichospermum* spp. were observed. These findings show that within a relatively small embayment, composition and toxin synthesis potential of cHABs can vary dramatically. This underscores the need for multifaceted management approaches and frequent cyanotoxin monitoring to reduce human health impacts.

## Graphical abstract

This work identified cyanobacterial community and cyanotoxin gene distribution in the Winam Gulf, Lake Victoria, Kenya. Cyanobacterial communities clustered in line with different areas of the Gulf, while cyanotoxin gene concentrations varied between the eastern and western regions. The results of the study show that the potential of toxin synthesis and cyanobacteria taxa present vary dramatically within the Gulf.

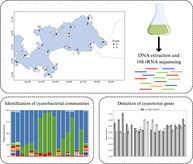

## INTRODUCTION

Cyanobacterial harmful algal blooms (cHABs) are a severe and growing threat to freshwater systems, affecting environmental and human health, fisheries, and recreation. Although considerable attention is paid to cHAB occurrence in North America (including the Laurentian Great Lakes) and Europe, their occurrence and impacts in the global south remain less well characterized. Specifically, Lake Victoria, which is shared by Kenya, Uganda, and Tanzania, is highly eutrophic and has in recent years experienced large toxic blooms of *Microcystis* spp. along with other cyanobacteria (Frank et al., 2023; Okello & Kurmayer, 2011; Olokotum et al., 2020, 2021, 2022). Cyanobacterial blooms are a lake‐wide issue but are especially prevalent in the bays and gulfs of Lake Victoria.

Lake Victoria is the second‐largest freshwater lake by surface area, and its basin has one of the highest population densities in Africa (Olokotum et al., 2020). Lake Victoria has several large and growing cities on its shores and the catchment is dominated primarily by cropland. Because of the rapid increases in population and agriculture in the area around Lake Victoria, eutrophication is an issue in the lake. Anthropogenic sources of excess nutrients are primarily from rivers that drain heavily farmed watersheds, sewage, and aquaculture operations, while atmospheric deposition of nitrogen (N) and phosphorus (P) from windblown burnt biomass is an additional contributor (Cheruiyot & Muhandiki, 2014; Olokotum et al., 2020). Nutrient dynamics in Lake Victoria have drastically changed as a result of land use change, and more recently, the reopening of the Mbita Channel (Njagi et al., 2022; Simiyu et al., 2022). Diatom communities have shifted to *Nitzschia* while cyanobacteria have proliferated over the past 50 years (Njagi et al., 2022). Urban area, farmland, and wetlands have increased while woodlands, forests, and grasslands decreased. As a result, total organic carbon, total nitrogen (TN), and P levels have increased in the lake, both in the water column and in sediments (Njagi et al., 2022).

Kenya's third largest city, Kisumu, is situated on the shore of Winam Gulf, and the city's drinking water comes from the Gulf. Along with drinking water for the city, nearshore fishing is a major economic industry in the region, as well as a food source for local subsistence fishers (Roegner et al., 2020). The lake also serves as a water source for drinking, laundry, and other household uses in many villages around the Gulf, where residents reported to use the water even if there was a thick visible green surface scum, many of which were producing microcystins (Obuya et al., 2023; Secaira Ziegler et al., 2022). Government officials in the region often campaign for practices to reduce exposure to toxins and eutrophication of the Gulf. These include treating or filtering water before drinking, avoiding bathing in the lake, and avoiding bringing livestock into the lake. Though these strategies would reduce risk of exposure to cyanotoxins, a lack of enforcement and infrastructure make implementation difficult in many communities (Secaira Ziegler et al., 2022). Children and other sensitive populations in these areas are especially susceptible to exposure to cHAB toxins. The region of Kenya around the Winam Gulf has high rates of HIV, malaria, and schistosomiasis; these diseases affect the immune system and lower body weight, increasing risk associated with long‐term exposure to microcystins (Roegner et al., 2020; Secaira Ziegler et al., 2022).

The Winam Gulf in Eastern Lake Victoria is shallow, with an average depth of 4.6 m (Gikuma‐Njuru et al., 2013). In a previous study that aimed to characterize phytoplankton community composition, it was found that the Gulf was dominated by *Microcystis* spp., with diazotrophic *Anabaena* present when N was limited later in the year (Sitoki et al., 2012). Both genera are microcystin producers, and this same study found microcystins above the WHO contact advisory standard, exceeding 100 μg L^−1^ (Codd et al., 2005; Sitoki et al., 2012). In the Gulf, P concentrations are lower than those observed in the open lake. Conversely, TN concentrations are lower in the open lake than Gulf due to active denitrification (Gikuma‐Njuru & Hecky, 2005). It is believed that soluble reactive phosphorus and total phosphorus (TP) from the open lake are contributing to the total P load in the Gulf (Gikuma‐Njuru & Hecky, 2005). Despite there being abundant sources of N from river outflow, TN:TP ratios in the Gulf are lower than the Redfield ratio, suggesting that the conditions are favourable for diazotrophic cyanobacteria (Gikuma‐Njuru & Hecky, 2005).

Although a cHAB may appear to be dominated by one or a few key cyanobacteria, there is potential for a variety of genotypes present within the community. Some genotypes of cyanobacteria that are of interest for monitoring efforts are those containing genes from the *mcy*, *sxt*, *cyr*, and *atx* operons encoding for production of microcystins, saxitoxins, cylindrospermopsins, and anatoxins, respectively (Cullen et al., 2019; Pearson & Neilan, 2008). Toxigenicity is not apparent when viewing the community through a microscope, but if the cyanobacteria contain the biosynthetic gene clusters there is potential for toxin production. Indeed, the environmental triggers for cyanotoxin production are largely unknown and differ for each cyanobacterial species (Paerl & Otten, 2013). This means that analysing the community for toxin‐production genes provides a basis for identifying risks and improving monitoring strategies to reduce exposure.

To our knowledge, there have been few studies characterizing water column bacterial communities through 16S rRNA gene amplicon sequencing over the entire Winam Gulf, particularly at regular limnological monitoring sites. Primarily, characterization of cyanobacteria has been done through morphological classifications (Lung'Ayia et al., 2000; Simiyu & Kurmayer, 2022; Sitoki et al., 2012). There are limited molecular studies that have been done in Lake Victoria or the Winam Gulf. Of the studies available, only one aimed to characterize bacterial communities in river outflows and wastewater treatment plant effluents (Wachira et al., 2022). Additionally, one dissertation aimed to characterize cyanobacteria and *mcyE* through denaturing gradient gel electrophoresis, and one study had a goal of characterizing *Microcystis aeruginosa* across East Africa using molecular techniques (Chhun, 2007; Haande et al., 2007). Other molecular studies looked at specific bacteria, such as *Escherichia coli*, *Vibrio cholerae*, and *Salmonella*, and this work generally centred around detections of these bacteria in fish (Awuor et al., 2011; Baniga et al., 2020; Hounmanou et al., 2019, 2022).

The aim of this work is to characterize bacterial and phytoplankton assemblages in the Winam Gulf through 16S and 18S rRNA gene sequencing. Through this, we can examine potentially toxin‐forming cyanobacteria in the Gulf and assess potential public health risks to the surrounding communities. A secondary goal of this work is to identify the potential of cyanotoxin production through quantitative polymerase chain reaction (qPCR). By sequencing the community, we can determine if there are regions of the Winam Gulf with a distinct bacterial and/or phytoplankton community structure. Additionally, we can find the toxin production potential of the cHAB and identify what species may be responsible for production of microcystins. Our hypothesis is that sites with distinct spatial characteristics (river inflow, nearshore, offshore) will have more similar bacterial communities to sites of the same type. However, we found that communities clustered by location in the Gulf rather than by landscape or hydrological features. We also found that communities were not dominated by *Microcystis* spp. and microcystins were generally in low to undetected concentrations, with exceptions to two sites. Furthermore, we identified evidence for a previously undetected cyanotoxin, cylindrospermopsin, that warranted additional sampling the following year.

## EXPERIMENTAL PROCEDURES

**FIGURE 1:**
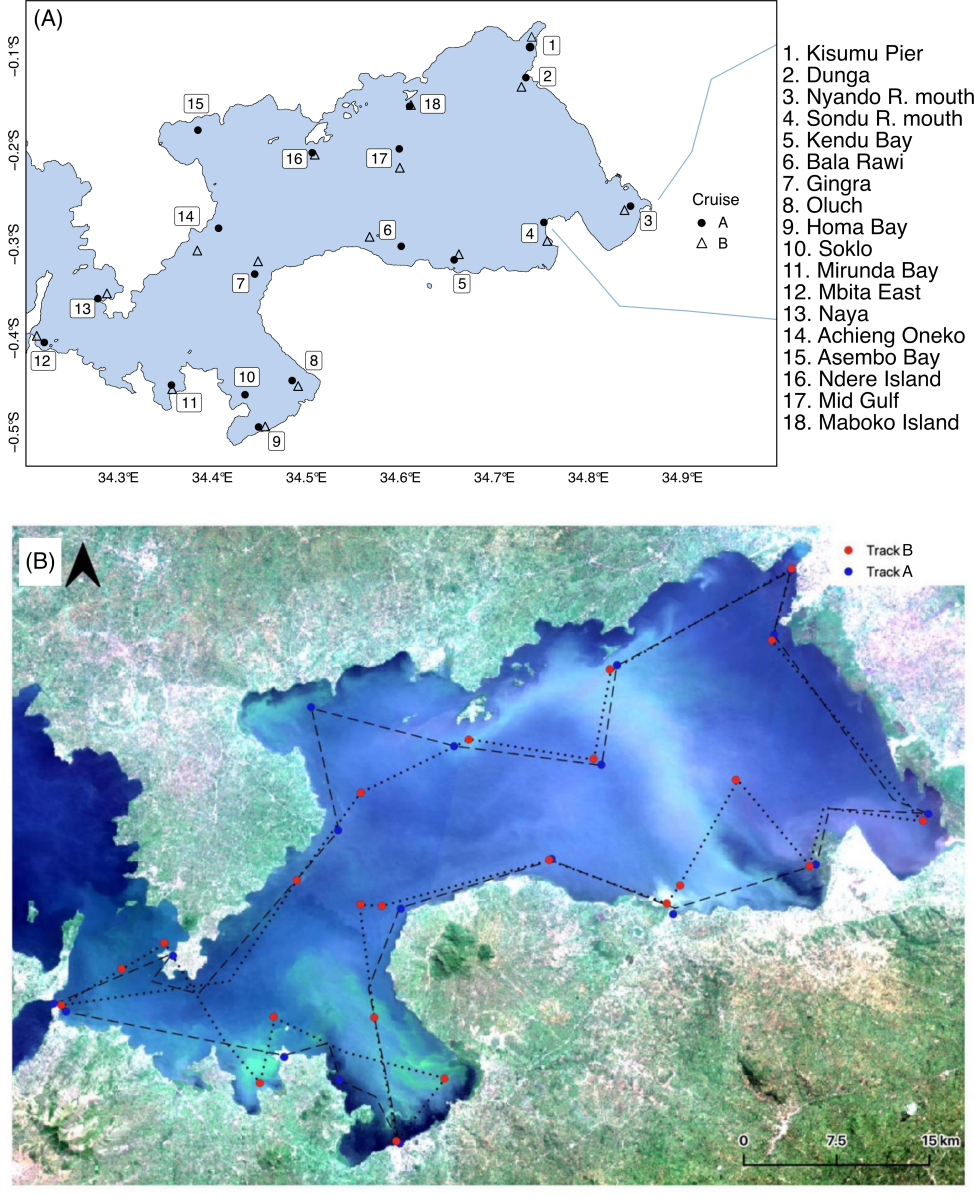
(A) Map of both cruise tracks. Sondu R. mouth and Nyando R. mouth are sites near the river mouth for the Sondu and Nyando respectively. (B) 2022 Cruise tracks A and B overlaid on a satellite image from the Gulf (taken 30 June 2022).

The study was conducted in the Winam Gulf, a shallow bay along the eastern shore of Lake Victoria. The Gulf is located entirely within Kenya, with a surface area of 1333 km^2^ (Gikuma‐Njuru et al., 2013). During the period of 22–25 June 2022 for cruise A, 18 sites were sampled off of R.V. *Uvumbuzi* throughout the Gulf (Figure 1A, B) The 2022 cruise B sampling occurred at 23 sites over 27–30 June 2022. All sites in cruise A, other than Soklo and Asembo Bay, were sampled during both cruises. In 2023, sampling occurred during the period of 29 May–3 June. For the purpose of this study, we define eastern bay sites as Sites 1–7 and 14–18, and western bay sites as Sites 8–13.

At each site, water was collected at 1 m depth three times using a 2.5 L Van Dorn sampler and mixed. A total of 1 m was chosen as the sampling depth as it was shallow enough for no sediment interference and minimal surface scums were reflective of a mixed water column. Microbial biomass was collected during cruise A by filtering water through Sterivex cartridge filter units (Sigma Aldrich, St. Louis, MO) with 0.22 μm pore size. Samples were preserved by filling cartridges with RNAlater solution (Invitrogen, Thermo Fisher Scientific Inc, Waltham, MA) and frozen at −20°C. At each site, an aliquot of lake water was also preserved with Lugol's iodine solution for identification of cyanobacteria and phytoplankton by microscopy. Physicochemical parameters (i.e., water temperature, dissolved oxygen, conductivity, total dissolved solids, pH) were measured using a multiparameter sonde (YSI Inc., Yellow Springs, OH).

Prior to DNA extraction, RNALater solution was flushed out of the Sterivex filter units with DNase‐free water (Invitrogen UltraPure DNase/RNase‐Free Distilled Water). Filter cartridges were opened using a pipe cutter and filter membrane was carefully removed. DNA from the filters was extracted using a DNeasy PowerWater kit (Qiagen, Germantown, MD) according to the manufacturer's protocol, and purity and quantity were assessed with a NanoDrop spectrophotometer (Thermo Fisher Scientific Inc.). Extracted DNA was kept frozen at −80°C until downstream analyses.

Quantification of cyanobacterial toxin gene abundances were assessed by quantitative PCR (qPCR) for cruise A using the multiplexed CyanoDTec total cyanobacteria and cyanotoxin gene kits (Phytoxigene, Akron, OH) and a Q‐qPCR thermocycler (Quantabio, Beverly, MA). Cycling conditions were selected according to Phytoxigene kit specifications. The total cyanobacteria kit amplifies the 16S rRNA gene of cyanobacteria using a universal primer set and contains an internal amplification control. The multiplexed cyanotoxin kit amplifies key genes involved in synthesis of microcystins/nodularins, saxitoxins, and cylindrospermopsins (*mcyE/ndaF*, *sxtA*, and *cyrA*, respectively) (Al‐Tebrineh et al., 2012).

Sequencing of 16S and 18S ribosomal RNA libraries for cruise A samples was performed at the University of Minnesota Genomics Center using a MiSeq sequencer (2 × 300 paired‐end reads; Illumina, San Diego, CA) and FastQC. Primer set 515F/809R was used to target the V4 region of the 16S rRNA gene for amplification (Gohl et al., 2016). The resulting FASTQ files were analysed in RStudio (version 2022.07.2) using a DADA2‐based workflow (version 1.22.0; Callahan et al., 2016). Sequences were trimmed to remove primers and low‐quality read ends, filtered (minimum quality score of 28), adjusted in accordance with the DADA2 error correction model, and paired‐end reads were merged (minimum overlap of 20 bases). From the merged paired‐end reads, an amplicon sequence variant (ASV) table was constructed and chimeric ASVs were removed. Taxonomy was assigned to ASVs using the SILVA 16S reference database (version 132) (Quast et al., 2012). The phyloseq package (v1.38.0) was used to generate relative abundance bar plots and hierarchical clustering analysis using Bray–Curtis dissimilarity (McMurdie & Holmes, 2013). Tidyverse (version 1.3.2) was also used for data visualization (Wickham et al., 2019). Spearman's rank was utilized for correlation analyses and forward selection analyses were performed for the microbial community, microcystins, and *mcyE* copies. One site, Kisumu Pier, was removed from 16S community analysis due to low read counts and quality scores. The 18S rRNA gene was amplified with primer set 1391F/EukBr targeting the V9 region (Amaral‐Zettler et al., 2009). The reads were also processed with the DADA2 workflow without merging paired‐end reads, and the Protist Ribosomal Reference database (version 4.14.1) was used for assigning taxonomy to the forward reads (Guillou et al., 2012).

Total microcystin concentration was measured in accordance with USEPA Method 546 for both cruises (U.S. EPA, n.d.). Briefly, whole water was collected at each of the 18 sites and then lysed using three cycles of freeze/thaw. The lysate was then assayed for microcystins using the Microcystins/Nodularins (ADDA) ELISA kit (Gold Standard Diagnostics, Warminster, PA) according to kit instructions. Absorbance was read at 450 nm using a Multiskan FC Microplate Photometer (Thermo Fisher Scientific Inc.). In 2023, cylindrospermopsins were measured using a Cylindrospermopsin ELISA kit (Gold Standard Diagnostics) in addition to microcystins.

To measure chlorophyll‐*a* for both cruise tracks, water was filtered onto 25 mm glass fibre filters (GF/F; Whatman, Maidstone, UK) in varying volumes depending on biomass concentrations and turbidity and placed into a 15 mL polypropylene centrifuge tube. Sample filters were frozen until processing when 5 mL of 90% acetone was added to the tube. Each sample was sonicated for 1 min before storage at 4°C in the dark overnight. Chlorophyll concentrations (μg L^−1^) were calculated by measuring absorbance (750, 665, 645, 630 nm) following a modified EPA Method 446 protocol with adjusted constants using a GENESYS 10 UV–Vis Spectrophotometer (Thermo Fisher Scientific Inc.) and adjusting to volume filtered (Arar, 1997).

Whole water was analysed by microscopy for phytoplankton abundance in a measured aliquot using the Utermöhl method with a magnification modification of the stratified counting technique of Munawar and Munawar (1976) as described in McKay et al. (2020).

## RESULTS

### Water quality variables

**FIGURE 2:**
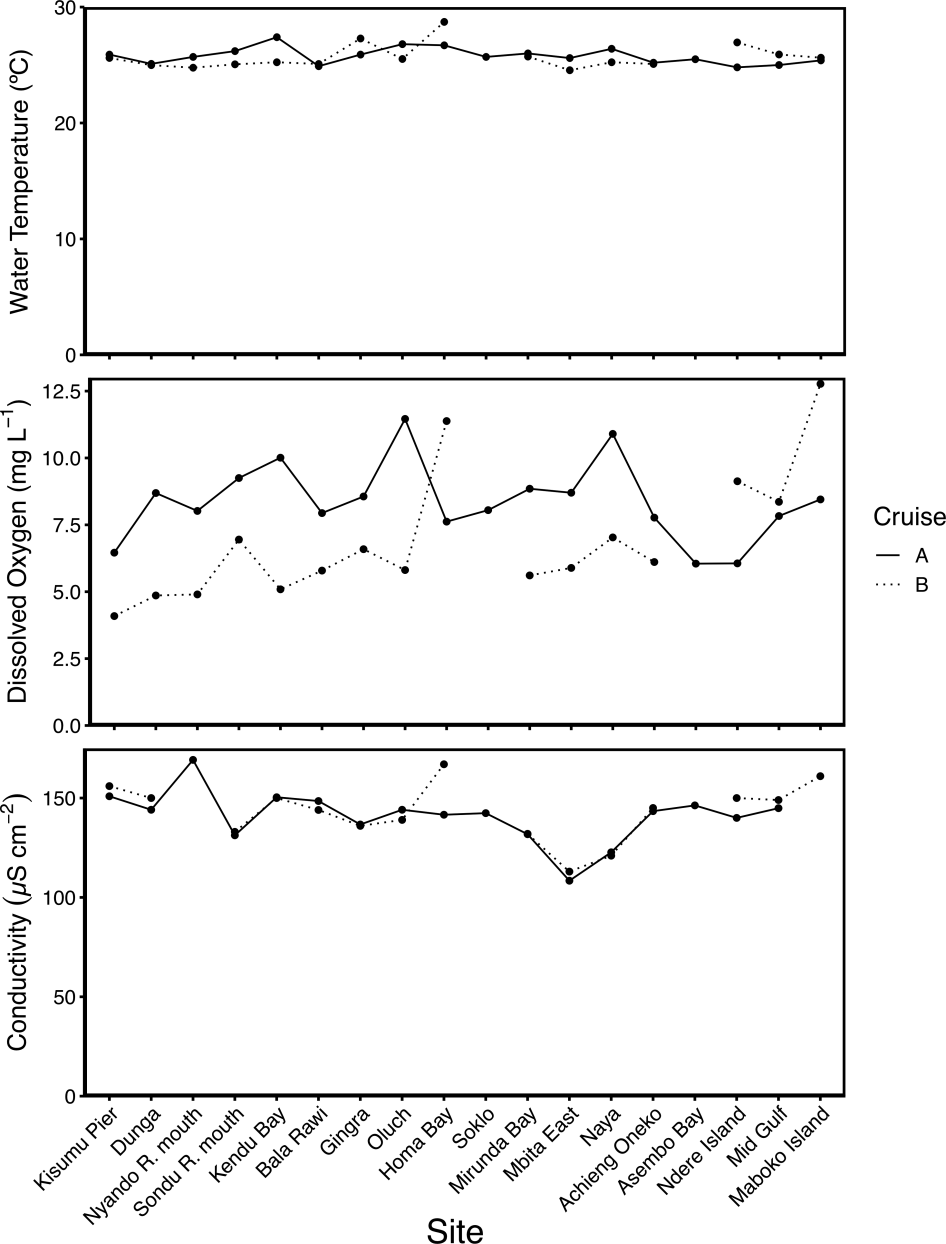
Water temperature (°C), dissolved oxygen (mg L^−1^), and conductivity (μS cm^−2^).

Water quality measurements were taken at each of the sites throughout the coastal areas of the Winam Gulf (Figure 2). Water temperature remained stable for sites on the cruise transect with a mean of 25.8°C, fluctuating four degrees Celsius during both cruises. Conductivity was consistent (143.1 ± 14.8 μS cm^−2^), besides two sites with the minimum (108.4 μS cm^−2^, cruise A) at Mbita East and maximum (179.0 μS cm^−2^, cruise B) at the Nyando R. mouth. In contrast, dissolved oxygen (DO; 7.68 ± 2.07 mg L^−1^) showed higher variability, with high values at Maboko Island (12.77 mg L^−1^, cruise B) and Oluch (11.46 mg L^−1^, cruise A) and low values at Kisumu Pier (4.09 mg L^−1^, cruise B) and Dunga (4.86 mg L^−1^, cruise B).

### *Chlorophyll‐*a *and microcystin concentrations*

Extracted chlorophyll‐*a* concentrations were measured as a proxy for phytoplankton biomass and were observed in moderate to high concentrations at sampling sites in the Gulf (Table 1). Near the Nyando R. mouth, chlorophyll‐*a* was in lower concentrations than in the embayments and offshore sites (11.69 ± 6.86 μg L^−1^ vs. 50.37 ± 79.29 μg L^−1^, respectively; *p*<0.05).

**TABLE 1:** Chlorophyll‐*a* concentrations of the Winam Gulf sampling sites. All sites other than Soklo and Asembo Bay include data from both cruises, *n* = 3.

Site name	Chlorophyll‐*a* (μg L^−1^)
Kisumu Pier	44.93 ± 11.90
Dunga	8.74 ± 4.54
Nyando R. mouth	11.69 ± 6.86
Sondu R. mouth	88.13 ± 78.11
Kendu Bay	21.51 ± 28.27
Bala Rawi	13.32 ± 7.68
Gingra	13.23 ± 9.55
Oluch	44.47 ± 26.69
Homa Bay	272.59 ± 172.00
Soklo	130.02
Mirunda Bay	27.60 ± 19.13
Mbita East	13.32 ± 9.78
Naya	19.81 ± 5.86
Achieng Oneko	19.51 ± 11.00
Asembo Bay	151.07
Ndere Island	69.52 ± 64.83
Mid Gulf	21.69 ± 10.55
Maboko Island	55.06 ± 30.98

Microcystin concentrations were analysed at all sites but were only detectable (≥0.15 μg L^−1^) at three sites: Mirunda Bay (0.15 μg L^−1^), Achieng Oneko (0.15 μg L^−1^), and Homa Bay (0.19 μg L^−1^) during cruise A. Despite the short time between cruises, microcystin analysis for cruise B revealed concentrations of 2.22 and 1.32 μg L^−1^ at Homa Bay and Mirunda Bay. Additionally, water taken from a pier along the shore of Homa Bay, as well as water from adjacent to the Homa Bay drinking water plant intake was assayed for microcystins. The intake was below the limit of detection, but the nearshore pier had the highest measured concentration of the entire cruise A survey with a concentration of 1.3 μg L^−1^. No apparent correlation between microcystins and chlorophyll‐*a* was observed (*r*_*s*_ = 0.15, *p* = 0.55). In contrast, analysis of cyanotoxins in 2023 revealed low but consistent concentrations of microcystins in the Gulf (0.3 ± 0.1 μg L^−1^), except for samples from the shore of Homa Bay and Kendu Bay where microcystins exceeded 5 μg L^−1^. Cylindrospermopsins were found in nonzero concentrations at Kisumu Pier, Dunga, and the Nyando R. mouth (estimated at 0.011, 0.035, and 0.003 μg L^−1^, respectively), but were outside the dynamic range of the assay for accurate quantification.

### Spatial patterns in bacterioplankton and chloroplast community composition

**FIGURE 3:**
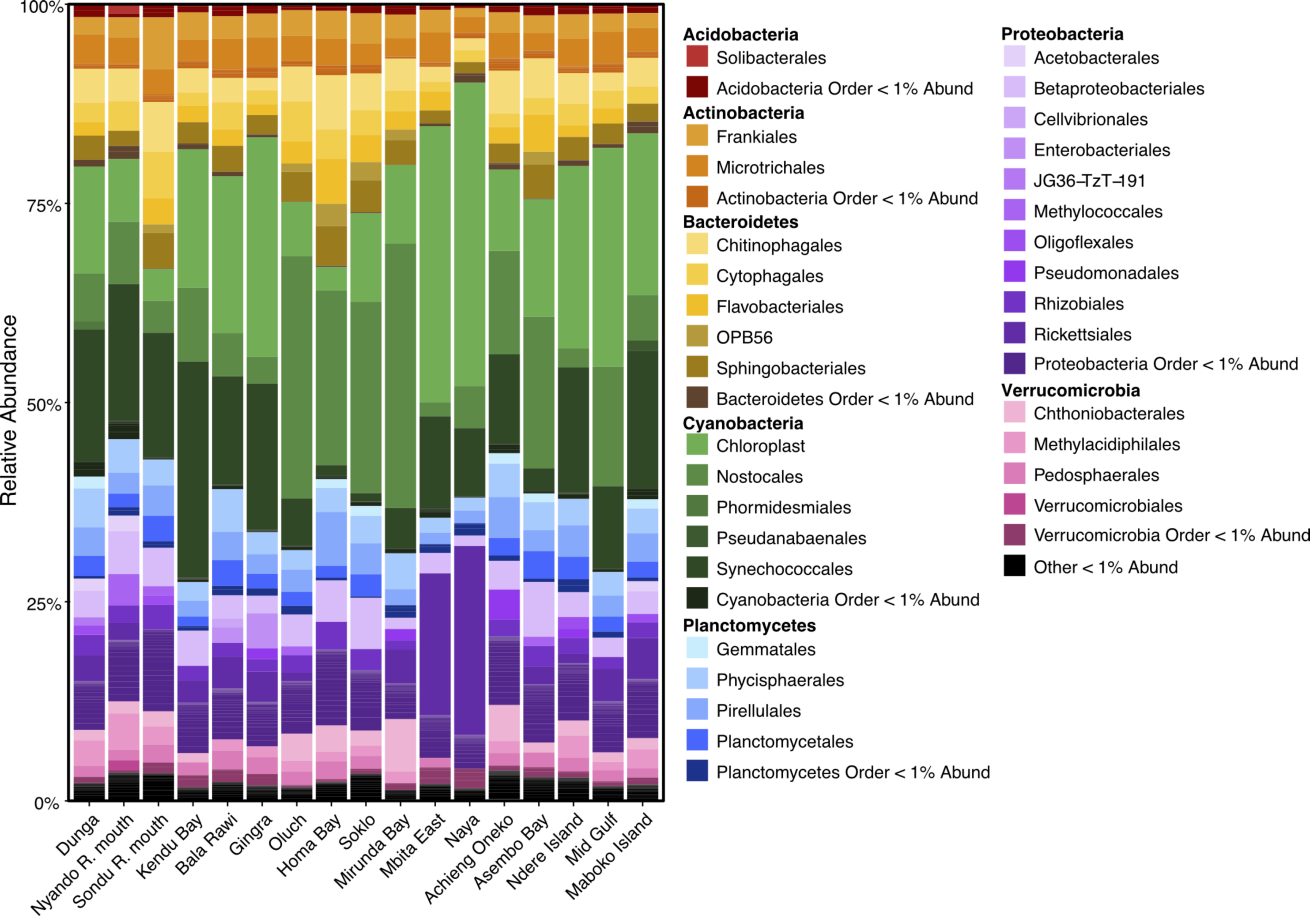
Relative abundance of prokaryotic orders. Orders below 1% abundance were aggregated at the phylum level.

Overall, the communities resembled those of other eutrophic freshwater lakes, with high relative abundances of Cyanobacteria, Bacteroidetes, Planctomycetes, Proteobacteria, Actinobacteria, and Verrucomicrobia (Figures 3 and S1; Gohl et al., 2016; Callahan et al., 2016; Quast et al., 2012; McMurdie & Holmes, 2013). Less abundant phyla Acidobacteria, Chloroflexi, and Latescibacteria were variable across the Gulf, not being present at all sites and never exceeding 5% at any one site. Dunga and Bala Rawi prokaryotic communities had the highest Shannon's diversity, while Naya had the lowest (S2a). The forward selection analysis on the microbial community revealed that of the measured environmental variables, total dissolved solids was the only the significant correlation (*p*<0.05).

While cyanobacteria were abundant throughout all sites constituting between 22% and 52%, communities varied between regions of the Gulf. Outer bay sites closest to the inlet to Lake Victoria, Mbita East, and Naya, had high abundances (34% and 38% respectively) of chloroplast sequences matching *Aulacoseira granulata var. angustissima* with 100% identity, while sites in the Homa Bay region (Oluch to Mirunda Bay) had higher Nostocales abundances (22%–33%) and Inner Bay sites (Dunga to Bala Rawi) had higher Synechococcales abundances (Figure 3). Despite the variation in photoautotrophic group relative abundances, the presence and proportions of orders within other phyla were more consistent across sites. Bacteroidetes orders Chitinophagales, Cytophagales, Flavobacteriales, and Sphingobacterales, often associated with freshwater organic matter degradation, were moderately abundant at all sites. At the sites Mbita East and Naya, the order Rickettsiales was substantially higher abundances of 17% and 23% respectively compared to other sites. Closer examination revealed that the sequences represented a putative obligate endoparasite, with 100% sequence similarity to *Candidatus* Megaira polyxenophila that infects freshwater *Paramecium*.

### Cyanobacterial assemblage distribution

**FIGURE 4:**
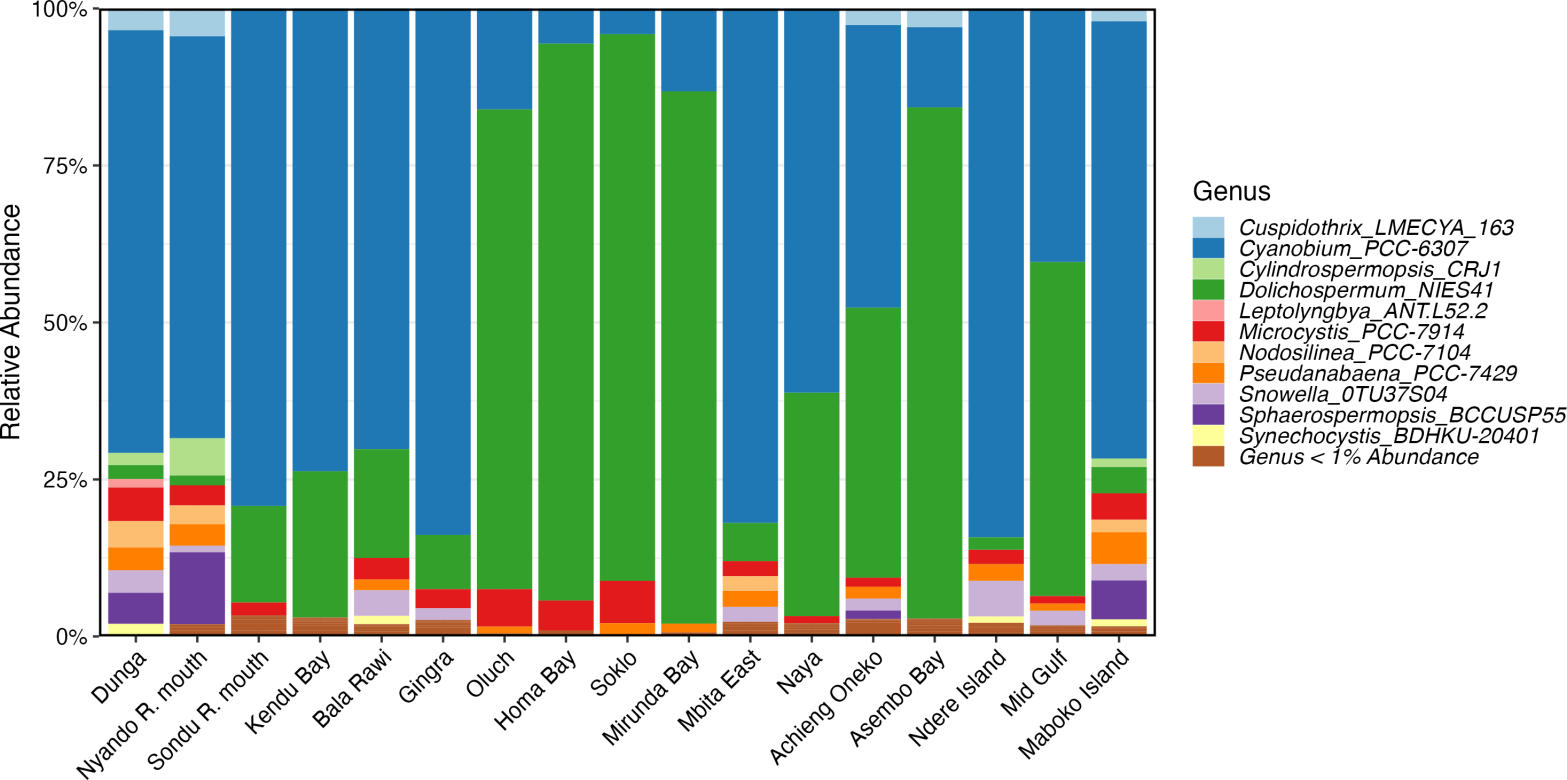
Relative abundance of cyanobacteria at the genus‐level.

Focusing on the cyanobacterial component of the communities, we found that *Cyanobium* spp. and *Dolichospermum* spp. were the dominant genera (Figure 4). We observed communities dominated by *Dolichospermum* spp. in the southernmost region of the western bay (sites 8–11; Figure 1) and northwestern region of the eastern bay (sites 14, 15, 17; Figure 1). *Microcystis* spp. were present in low abundance (0.2%–6.7%), and *Pseudanabaena* spp. co‐occurred with *Microcystis* in many samples. Dunga, Nyando R. mouth, Kendu Bay, and Mid Gulf cyanobacterial assemblages had the highest Shannon's diversity (Figure S2b). Though observed in previous years, *Cylindrospermopsis* spp. were only found in abundances greater than 1% at three sites (Nyando R. mouth, Dunga, and Maboko Island) in the northeastern portion of the Gulf (Sitoki et al., 2012). The ASVs associated with *Dolichospermum* throughout the Gulf were most related to *Dolichospermum flos‐aquae* PMC206.03 isolated from Senegal, with percent identities ranging from 99.6% to 100% (Duval et al., 2018).

### Microscopy of cyanobacteria and eukaryotic phytoplankton

**FIGURE 5:**
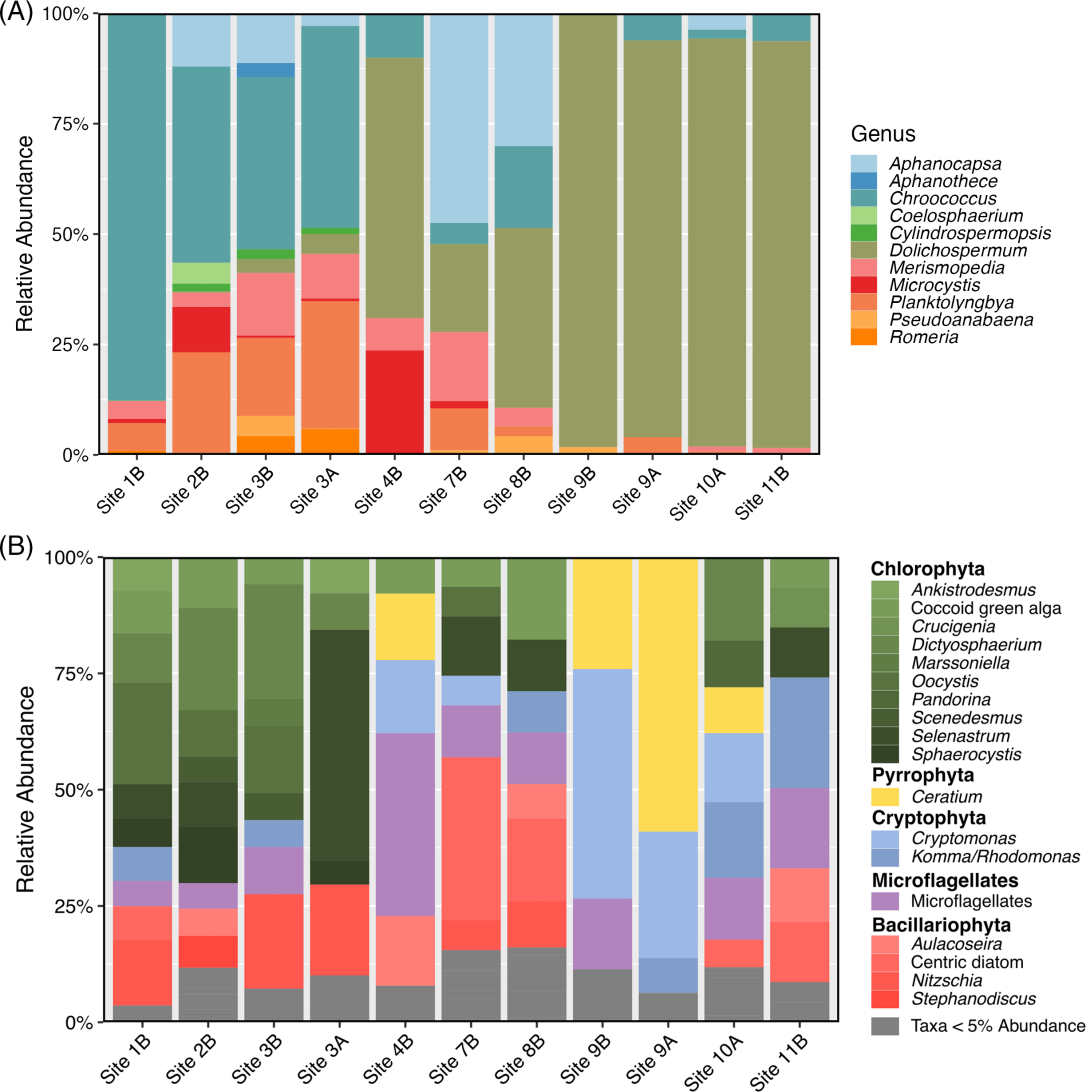
Microscopy counts of (A) cyanobacterial genera and (B) eukaryotic phytoplankton. Taxa below 5% abundance were aggregated at the phylum level.

Through microscopy, we saw a similar distribution of cyanobacterial and eukaryotic phytoplankton as compared to the sequencing results from cruise A (Figure 5A, B). Analysis found that picocyanobacteria (*Aphanocapsa*, *Aphanothece*, *and Chroococcus*) were the primary cyanobacterial genera and Chlorophyta (green algae) were the dominant eukaryotic phytoplankton in the northeastern sampling sites. In the Homa Bay region, *Dolichospermum* were the prominent cyanobacteria and the eukaryotic phytoplankton distribution was distinct from other sites. At all sites other than Homa Bay (site 9), Bacillariophyta (diatoms) were present in abundance over 5%. Samples analysed from both cruise A and B (Nyando R. mouth, 3; Homa Bay, 9) showed minor differences in microscopy for the eukaryotic phytoplankton. At the Nyando R. mouth, cruise B samples contained *Cryptomonas* and microflagellates, while these taxa were absent in cruise A samples. Similarly, the cruise A sample from Homa Bay did not contain microflagellates.

### Cyanotoxin gene detections

**FIGURE 6:**
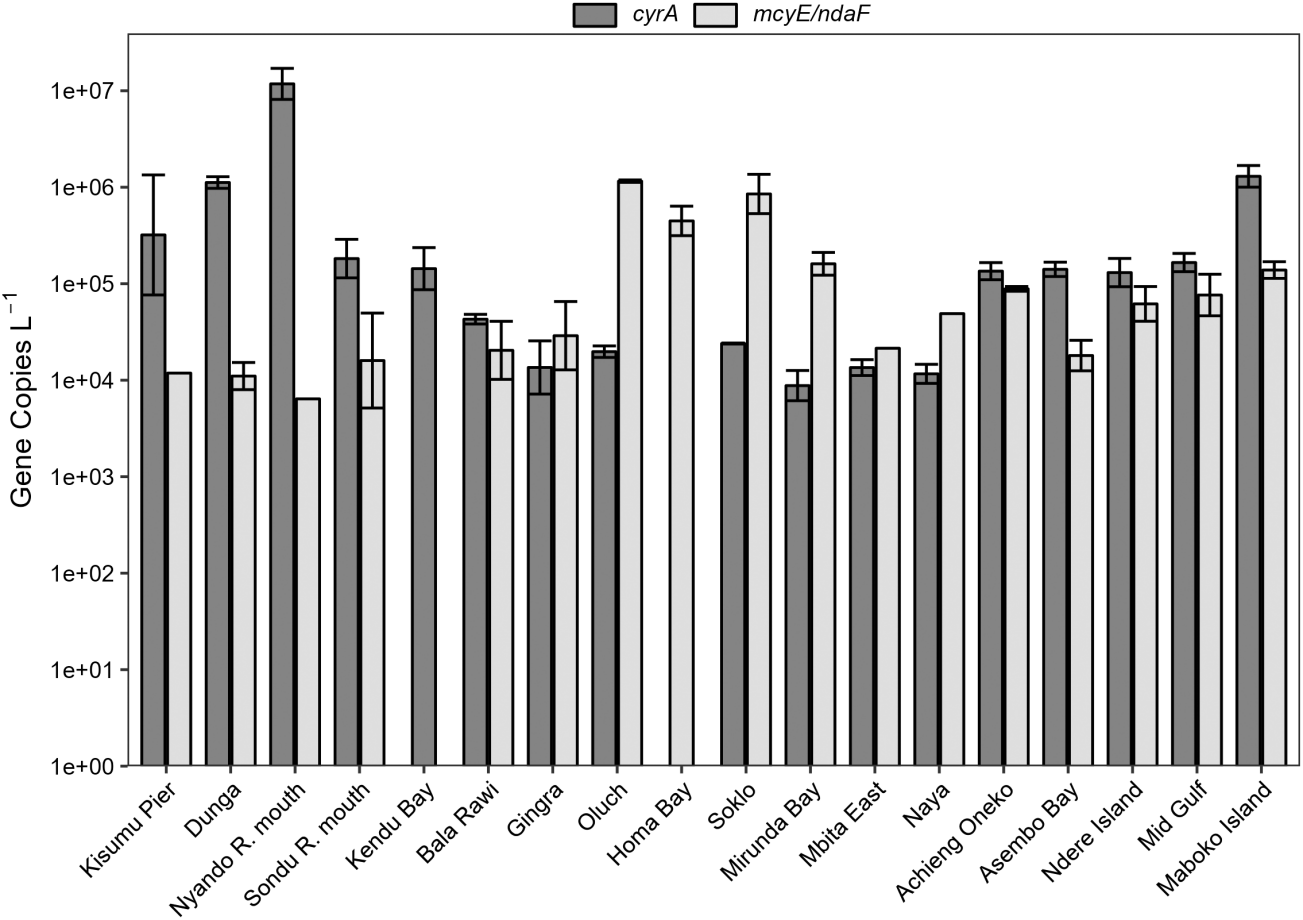
Cyanotoxin synthetase gene (*mcyE/ndaF, cyrA*) detections in the Winam Gulf sampling sites during the 2022 cruise. *Y*‐axis is on a log scale. Error bars represent standard deviation of *n* = 3.

By analysing the cyanotoxin gene copies, we observed that both *cyrA* and *mcyE/ndaF* copies were found at nearly every site (Figure 6), while *sxtA* genes were not detected in any samples. Across the entire cruise track, *mcyE/ndaF* detections ranged from 6.4 × 10^3^ (Nyando R. mouth) to 1.2 × 10^6^ (Oluch) copies L^−1^ and *cyrA* detections ranged from 9.2 × 10^3^ (Mirunda Bay) to 1.2 × 10^7^ (Nyando R. mouth) copies L^−1^. No *mcyE/ndaF* copies were detected at Kendu Bay and no *cyrA* copies were detected at Homa Bay. In 11 of the 18 sites analysed, *cyrA* copies were found in higher abundance than *mcyE/ndaF*. At the sampling sites Oluch, Homa Bay, and Soklo, *mcyE/ndaF* copy concentrations were higher in comparison to all other sites. At Nyando R. mouth, *cyrA* gene copies were significantly higher than any other site, with 1.2 × 10^7^ copies L^−1^ detected (*p* = 0.02).

In 2023, *cyrA* copies were found in lower concentrations at Kisumu Pier, Dunga, and Nyando R. mouth corresponding with the detection of cylindrospermopsins. Concentrations were 4.5 × 10^5^, 1.1 × 10^5^, and 3.9 × 10^4^ copies L^−1^, respectively.

### Eukaryotic community distribution

**FIGURE 7:**
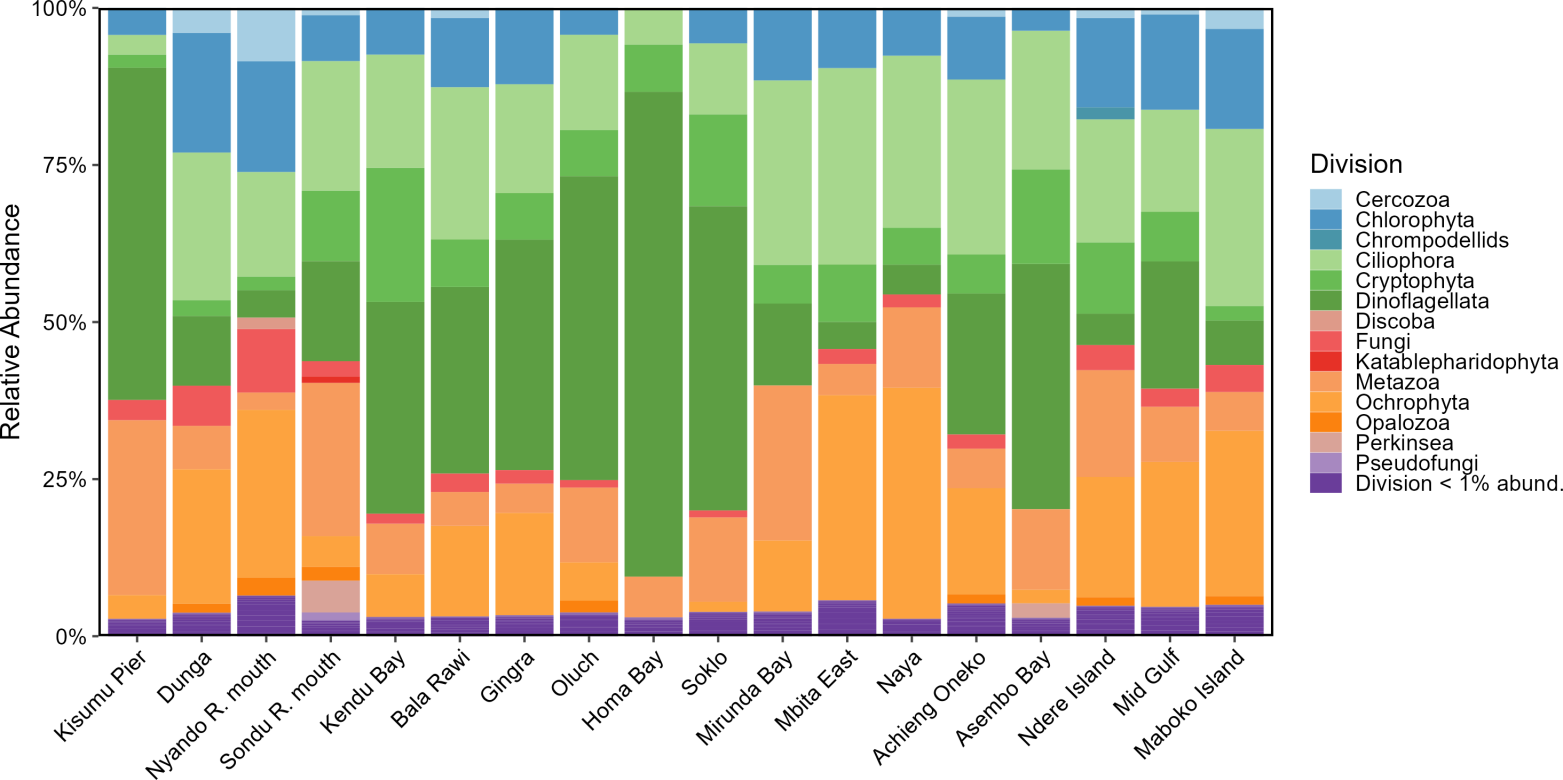
Relative abundance of eukaryotes at the division level.

By examining the eukaryotic communities within the Winam Gulf, we were able to characterize and determine the distribution of major groups of photosynthetic eukaryotes (Figure 7). Distributions of eukaryotic divisions varied widely across sites with some discernable patterns. For example, dinoflagellates were in high abundance at many sites in the southern portion of the outer bay and near Homa Bay, with their abundance highest at Homa Bay (78%). Dinoflagellate communities were primarily comprised of *Ceratium*. Additionally, we observed that when dinoflagellate abundances were higher, Ochrophyta (belonging to the stramenopile group, inclusive of diatoms) abundances were generally lower and vice versa (*r*_*s*_ = −0.88, *p*<0.05). Chlorophyta and stramenopiles were also found at all sites in greater than 1% abundance, except Homa Bay, with average abundances across the Gulf of 10% ± 5% and 15% ± 11%, respectively. Relative abundance of diatoms exceeded 30% at two sites: Mbita East (33%) and Naya (37%), which are closest to the Rusinga Channel.

Although in relatively low overall abundance (2.67% ± 2.42%), fungi were present in greater than 1% abundance at many sites, except Homa Bay, Mirunda Bay, and Asembo Bay. Relative abundance was highest at the Nyando R. mouth (10%) and lowest at Asembo Bay (0.03%). In a few cases, sites with higher abundances of stramenopiles had higher abundances of fungi (*r*_*s*_ = 0.59, *p*<0.05). At Dunga, relative fungi abundance was 6% and stramenopiles was 22%, and at Nyando R. mouth abundances were 10% and 28% for fungi and stramenopiles, respectively.

### Clustering of sites through community similarities

**FIGURE 8:**
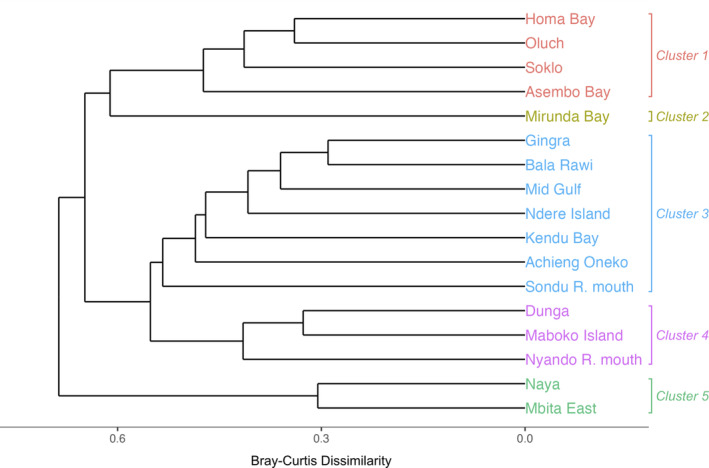
Hierarchical clustering of sampling sites based on compositional similarities of prokaryotic communities.

Given the observed spatial patterns in prokaryotic and eukaryotic communities, we sought to identify distinct geographical clusters based on similarities in community compositions between sites. Using hierarchical clustering of prokaryotic communities, we found that sites clustered into three distinct branches, with 5 total clusters, arranged mostly in line with the sites' geographical location within the Gulf. The three branches generally represented the sites near the Rusinga Channel (Naya, Mbita East; Cluster 5), within/near Homa Bay (Homa Bay, Oluch, Soklo, Asembo Bay; Cluster 1), Mirunda Bay (Cluster 2), and the eastern Gulf, including the remaining sites grouped together (Figure 8). The exception to the geographical clustering trend was Asembo Bay, which was found in Cluster 1 with Homa Bay. The pairs Homa Bay and Oluch were most similar to one another (Bray–Curtis dissimilarity = 0.34) as well as Gingra and Bala Rawi (0.29). Dunga and Maboko Island were most related in another cluster (0.33), and most distant from the other sites was the pair Naya and Mbita East (0.31). Interestingly, the location within the Gulf appears to have a greater influence on community similarities than landscape features or hydrological characteristics, such as proximity to river mouths, as these sites did not cluster together. In contrast to the four well‐defined groups, Mirunda Bay was clustered alone.

## DISCUSSION

In this initial study of the Winam Gulf, we generated a dataset inclusive of all bacterioplankton with a focus on cyanobacteria and characterization of photosynthetic eukaryotic communities. Additionally, we were interested in the spatial distribution of cyanotoxins and toxigenic cyanobacteria. For both prokaryotic and eukaryotic communities, differences were noted that were aligned with their location in the Gulf, suggesting that localized physicochemical and/or unmeasured limnological factors were important in structuring the communities. Within the cyanobacteria, taxa associated with N‐fixation were predominant, whereas *Microcystis* was only a minor part of the community. Microcystins were not frequently detected; however, toxigenic cyanobacteria were present at every site.

### Toxigenic cyanobacteria were present, but not dominant

Prior to our study, *Cyanobium* spp. abundance in the Winam Gulf was not well described in the literature. *Cyanobium* spp., part of the *Synechococcus* collective, were the dominant cyanobacteria at most sites (Salazar et al., 2020). At a few sites, *Cyanobium* spp. and *Dolichospermum* spp. were found at similar relative abundances. These cyanobacteria have co‐occurred in previous systems since *Cyanobium* spp. is efficient at N acquisition and *Dolichospermum* spp. can fix N (Li et al., 2020). In Lake Erie, *Cyanobium* and *Synechococcus* OTUs are described in 16S rRNA gene sequencing data, but microscopy often does not allow for resolution beyond picocyanobacteria (Berry et al., 2017; Matson et al., 2020; Ouellette et al., 2006). We saw that through microscopy, the genera of picocyanobacteria identified differed from sequenced samples where ASVs were assigned to *Cyanobium*. Identification of picocyanobacteria can be difficult due to size, but databases for assigning taxonomy may also be limited and there is no complete consensus between morphology and 16S rRNA gene sequences (Jasser & Callieri, 2016; Yarza et al., 2014). The variability in taxonomic assignments from these samples emphasizes the importance of both traditional microscopy and sequencing approaches.

*Microcystis* spp. has been traditionally recognized as a concern in the Winam Gulf, however, at time of sampling it was not a prominent part of the community (Sitoki et al., 2012). The low abundance of *Microcystis* spp. may be attributed to recent changes in water flow due to the removal of the Mbita causeway blocking flow from the open waters of Lake Victoria (Simiyu et al., 2022). Replacement of the causeway with a bridge has lowered conductivity and has been linked to increased diatom abundance and decreased *Microcystis* populations (Simiyu et al., 2022). At most, during our study, *Microcystis* spp. was ~7% of the community at Soklo. In microscopy samples from cruise B, we observed a higher relative abundance at the Sondu R. mouth as compared to what was found through sequencing from cruise A. Despite the short amount of time between cruises, the *Microcystis* spp. community increased in density due to growth and/or movement of the bloom, but due to lack of continuous satellite imagery, we are unable to determine the exact factor contributing to the increase in abundance. There was an apparent association between *Microcystis* spp. and *Pseudanabaena* spp. presence (*r*_*s*_ = 0.45, *p* = 0.07). The small, cosmopolitan cyanobacteria *Pseudanabaena* spp. is often associated with *Microcystis* spp. colonies (Agha et al., 2016; Smith et al., 2021). *Pseudanabaena* spp. typically grows within the mucilage of *Microcystis* spp. colonies and this association is likely due to regulation of light levels and access to nutrients (Agha et al., 2016).

Though the bloom was not actively producing microcystins when cruise A samples were collected, it was found to be toxigenic, with *mcyE/ndaF* detected at all sites except Kendu Bay. Therefore, the cyanobacteria bearing these toxin genes could initiate microcystin production under favourable conditions (Beversdorf et al., 2015). In contrast, cruise B found elevated microcystin concentrations in the Homa Bay region, but *Microcystis* spp. was not identified in either cruise microscopy samples. As *Microcystis* spp. was in low relative abundance in the sequencing data, there is potential that this small community was not captured by microscopy. The World Health Organization (WHO) has guidelines for exposure to cHAB blooms based on chlorophyll‐*a* levels with increased risk of health effects above 50 μg L^−1^ (Codd et al., 2005). Chlorophyll‐*a* levels at many sites in the Winam Gulf greatly exceeded WHO thresholds, yet microcystin concentrations were primarily below detection, with the exception of the Homa Bay Pier, which was slightly higher than WHO guidelines of 1.0 μg L^−1^ for microcystins in drinking water.

We suspect that *Microcystis* spp. were responsible for the majority of the *mcyE/ndaF* gene detections, as they generally followed trends in *Microcystis* spp. abundance rather than *Dolichospermum* spp. Moreover, we isolated a toxigenic *Microcystis panniformis* from water sampled near Homa Bay, further supporting that *Microcystis* spp. may be contributors of microcystin synthetase gene copies (Brown et al., 2024).

### First detection of the cylindrospermopsin synthetase gene in the Winam Gulf

Although identified previously in the Gulf, *Cylindrospermopsis* spp. was not recognized as a cyanobacterial genus of high concern (Sitoki et al., 2012). In this study, *Cylindrospermopsis* spp. was detected in all sites, but in about half of the sites, abundance was extremely low within the community. At the site with the highest abundance of *Cylindrospermopsis* spp., the Nyando R. mouth, there was no visible surface bloom and there was no association between higher turbidity and abundance of *Cylindrospermopsis* spp. (*r*_*s*_ = −0.004, *p* = 0.9894). At the river mouth, sample water was turbid and had a large amount of visible sediment. However, *Cylindrospermopsis* spp. blooms can be facilitated by increases in turbidity, as it is adapted to a broad range of light conditions (Burford et al., 2016; Wood et al., 2014). The 16S detections of *Cylindrospermopsis* spp. at Nyando R. mouth were confirmed through microscopy, where 6300 cells mL^−1^ were counted.

Detection of the *cyrA* gene at nearly all sampling sites was unexpected. Cylindrospermopsins have been measured in open Lake Victoria in minimal concentrations (0.004–0.01 μg L^−1^), far below WHO drinking water guidelines, but we have not encountered any reports of this cyanotoxin in the Winam Gulf (Geneva: World Health Organization, 2020; Mchau et al., 2021; Omara et al., 2023). There is some potential that other cyanobacteria detected (*Cuspidothrix* spp. and/or *Dolichospermum* spp.) make up part of the *cyrA*‐containing population but given the covarying trends in *Cylindrospermopsis* spp. abundances and *cyrA* copy concentrations, it is likely that *Cylindrospermopsis* is primarily responsible (Geneva: World Health Organization, 2020). This is best illustrated at the Nyando R. mouth, where relative abundance and *cyrA* copies were both highest (6% and 1.2 × 10^7^, respectively). Previously, it was found that *C. raciborskii* AWT205 contained 1.13 ± 0.19 copies of *cyrA* per cell (Al‐Tebrineh et al., 2012). In our study, with 1.2 × 10^7^ *cyrA* copies L^−1^and a population of 6.3 × 10^6^ cells L^−1^, each cell would have about two copies of *cyrA*, assuming all *Cylindrospermopsis* spp. cells were toxigenic. This suggests that the dominant Winam Gulf strains have more *cyrA* copies per cell than AWT205, so *Cuspidothrix* and *Dolichospermum* may also be contributing to the total.

Both *Cylindrospermopsis* spp. and cylindrospermopsins (therefore *cyrA*) are common in tropical regions. While *Cylindrospermopsis* spp. and *cyrA* genes have been detected in a number of African surface waters, it appears that distribution of cylindrospermopsins themselves is understudied (Kokociński et al., 2017; Moreira et al., 2021; Scarlett et al., 2020). These novel detections provide evidence of potential cylindrospermopsin production in the Winam Gulf and can help to improve future cyanotoxin detection efforts. This also provides support for informing the public that lake water appearing to have no bloom may contain cyanobacteria and toxins. However, despite there being regulations for cylindrospermopsins and other cyanotoxins in drinking water, many communities surrounding the Gulf and in the global south as a whole do not have consistent access to processed drinking water. One previous survey found that about 50% of the surveyed group from Winam Gulf communities had no options other than using raw lake water (Roegner et al., 2020). Therefore, these guidelines in this region may often be of little importance to many people and are overall more useful to communities that have consistent and widespread availability of treated drinking water.

### Spatial distribution of prokaryotic and eukaryotic communities

Over both sampling periods, the physicochemical parameters were consistent throughout the Winam Gulf, with the exception of DO. The fluctuations in DO concentrations could have been a result of varying rates of photosynthesis at different times of day when sampling occurred. Sites with high and low conductivity correspond to two notable locations, the Nyando R. mouth and the Rusinga Channel. As the Nyando River flows through fertilized farmland and tea plantations, accumulating solutes from runoff likely contribute to higher conductivity waters entering the Gulf (Raburu & Okeyo‐Owuor, 2006; Gabellone et al., 2005). Conversely, the conductivity of open Lake Victoria is low, and through mixing yielded the lower conductivity observed at Mbita East, as previously found in the Winam Gulf (Simiyu et al., 2022; Sitoki et al., 2010). Overall, with the exception of these locations, this indicates that the Gulf is well‐mixed and only few distinct water masses are present, where water chemistry differences might be a key determinant of microbial community composition.

To view how diversity and abundances were related among sites, we used Bray–Curtis dissimilarity to cluster sites together based on the entire community. For prokaryotic communities, we observed that in the sites surrounding Homa Bay (Cluster 1), the order Nostocales was in relatively high abundance (22%–33%). Asembo Bay was in Cluster 1 as well because of a *Dolichospermum* spp. dominated community. Similarly, sites with greater than 1% *Cylindrospermopsis* spp. abundance and high *cyrA* copy concentrations were also clustered together (Cluster 4). Due to the similarities of other bacterial phyla at the sampling sites, the Bray‐Curtis clustering most closely follows trends in cyanobacterial genera.

While cyanobacteria were responsible for the Winam Gulf bloom, eukaryotic phytoplankton were major contributors. Several photosynthetic eukaryotes were found by analysing the 18S rRNA, including dinoflagellates and diatoms (Ochrophyta). We observed similar trends of high dinoflagellate abundance at Kisumu Pier and Homa Bay, but not at Sondu R. mouth (Kundu et al., 2017; Lung'Ayia et al., 2000). Diatoms have been historically found in Lake Victoria and the Winam Gulf, but as stated prior, diatom communities have shifted as a result of eutrophication (Njagi et al., 2022). However, the two sites near the Rusinga Channel had higher abundances of diatoms and this is likely a direct result of water exchange from the open lake and is indicative of less eutrophic waters (Simiyu & Kurmayer, 2022). This is further supported by the high abundance of chloroplast reads in these two sites that classified to Bacillariophyta. While less abundant overall, Chlorophyta and Cryptophyta were always present and sometimes exceeded relative abundances of the other eukaryotic phytoplankton.

Along with eukaryotic phytoplankton, we were interested in finding potential parasites that may be correlated with these communities. Within the true fungi community are many taxa known to be parasitic, and while these communities are of low abundance it is worth pointing out that several of these species are known to infect diatoms. Many of the most abundant fungi that are parasitic belong to the phylum of Chytridiomycota (chytrids). Within the chytrids are the genera *Zygophlyctis* (formerly *Zygorhizidium*), and these genera are known to parasitize diatoms *Aulacoseira* and *Ulnaria* (formerly *Synedra*), both of which were found within the Gulf (Seto et al., 2020). Mbita East, Naya, and Nyando R. mouth had the highest relative abundances of diatoms but only the Nyando R. mouth had high abundances of fungi. Since Mbita East and Naya are located in the channel, it is likely that these waters are turbulent from seiche activity. Consequently, such water movement could be limiting chytrid pathogenesis, as described by McKindles et al. (2021) and Wagner et al. (2023).

## CONCLUSIONS

Above we described characterization of bacterial and eukaryotic communities of the Winam Gulf, Kenya, and the first detections of cylindrospermopsin synthetase genes (*cyrA*) indicating potential for cylindrospermopsin production. We primarily found that bacterial communities at a high level were similar among all sites and that the cyanobacterial communities were diverse having different dominant genera between sites. As microcystins have been the most highlighted cyanotoxin of concern in the Gulf, finding potential for cylindrospermopsin production emphasizes the need for broader cyanotoxin monitoring (Plisnier et al., 2022).

Many people using lake water are aware that green water or a surface scum may be harmful to use and contain cyanotoxins, but at the site with highest *cyrA* copy concentration (Nyando R. mouth), no bloom was visibly apparent (Obuya et al., 2023; Secaira Ziegler et al., 2022). The risk of cyanotoxin consumption is high in these cases where the cyanobacteria may be distributed throughout the water column and obscured by suspended sediment. The triggers for cyanotoxin production are also not well‐characterized, with differential effects from temperature, nutrients, and light that are often different between cyanobacterial genera (Chaffin et al., 2018; Dyble et al., 2006; Mesquita et al., 2019; Paerl & Otten, 2013; Wagner et al., 2021). Additionally, the triggers for cyanotoxin production in the Gulf and other African waterbodies may differ from North American and European waterbodies, where many of these studies are done.

We suggest further research into cyanotoxin production in the Gulf, from the most abundant cyanobacteria present. We also suggest continual monitoring of bacterial communities to describe how communities change over the year, and between years. Ongoing monitoring will also result in stronger environmental datasets for analysis alongside community data. Continuing studies are warranted to determine how communities and cyanobacterial dominance changes between the rainy and dry seasons, potentially as a result of changing nutrient runoff. Most important of all, regular monitoring of cyanotoxins in surface waters and drinking water intakes could reduce exposures to those using raw lake water.

## AUTHOR CONTRIBUTIONS

**Katelyn M. Brown:** Conceptualization (equal); data curation (equal); formal analysis (lead); investigation (equal); methodology (equal); software (equal); validation (equal); visualization (equal); writing – original draft (lead); writing – review and editing (equal). **Katelyn B. Barker:** Conceptualization (equal); data curation (equal); investigation (equal); methodology (equal); validation (equal); writing – original draft (supporting). **Ryan S. Wagner:** Conceptualization (equal); data curation (equal); formal analysis (supporting); investigation (equal); methodology (equal); software (equal); validation (equal); visualization (equal); writing – original draft (supporting). **Christopher S. Ward:** Visualization (equal); writing – original draft (supporting); writing – review and editing (equal). **Lewis Sitoki:** Conceptualization (equal); writing – review and editing (equal). **James Njiru:** Conceptualization (equal); writing – review and editing (equal). **Reuben Omondi:** Conceptualization (equal); writing – review and editing (equal). **James Achiya:** Conceptualization (equal); resources (equal); writing – review and editing (equal). **Albert Getabu:** Conceptualization (equal); writing – review and editing (equal). **R. Michael McKay:** Conceptualization (equal); funding acquisition (equal); supervision (equal); writing – review and editing (equal). **George S. Bullerjahn:** Conceptualization (equal); funding acquisition (equal); supervision (equal); writing – review and editing (equal). **NSF‐IRES Lake Victoria Research Consortium:** Funding acquisition (equal); resources (equal).

## CONFLICT OF INTEREST STATEMENT

The authors declare no conflict of interest.

## Supporting information


**Supplemental Table 1.** Microcystin concentrations (μg L^−1^) in 2022 of sampling sites for both cruises, Homa Bay drinking water plant intake, and Homa Bay pier.

**Supplemental Figure 1.** Relative abundance of prokaryotes at the phylum level.

**Supplemental Figure 2.** (A) Prokaryotic alpha diversity. (B) Cyanobacteria‐only alpha diversity.

## Data Availability

The data that support the findings of this study are openly available in GenBank (https://www.ncbi.nlm.nih.gov/) under the BioProject reference number PRJNA1023928.

## APPENDIX A

**2022 NSF‐IRES Lake Victoria Research Consortium Participants**: Dorine Achieng^4^, George M. Basweti^4^, Max Beal^8^, Aidan Byrne^9^, William R. Cody^10^, Ken G. Drouillard^7^, Linet I. Kiteresi^4^, Theodore Lawrence^!^, Davide Lomeo^9^, Jared B. Miruka^4^, Samantha Mohney^#^, Kaela Natwora^$^, Julia A. Obuya^4^, Pamela Okutoyi^&^, Mark Olokotum*, Dennis Otieno^4^, Kefa M. Otiso^!!^, Omondi A. Owino^##^, Winnie Owoko^4^, Bethwell Owuor^5^, Anakalo Shitandi^5^, Jordyn Stoll^$$^, Mariam N. Swaleh^&&^, Emma Tebbs^9^, Emily Varga^7^, Brittany N. Zepernick**. ^8^University of Wisconsin‐Madison, Madison, Wisconsin, USA, ^9^Department of Geography, King's College London, United Kingdom, ^10^Aquatic Taxonomy Specialists, Malinta, Ohio, USA, ^!^African Center for Aquatic Research and Education, ^#^George Mason University, Fairfax, Virginia, USA, ^$^University of Minnesota‐Duluth, Duluth, Minnesota, USA, ^&^Kenya Climate Innovation Centre Consulting, Nairobi, Kenya, *National Fisheries Resources Research Institute, Makerere University, Jinja, Kampala, Uganda, ^!!^School of Earth, Environment and Society, Bowling Green State University, Bowling Green, OH, USA, ^##^Department of Medical and Applied Sciences, Sigalagala National Polytechnic Kakamega, Kenya, ^$$^Kent State University, Kent, Ohio, USA, ^&&^Technical University of Mombasa, Mombasa, Kenya, **University of Tennessee Knoxville, Knoxville, Tennessee, USA.
