# Supplementary material for: Bacterial community and cyanotoxin gene distribution of the Winam Gulf, Lake Victoria, Kenya
Source: Environ Microbiol Rep. 2024 Jun 17;16(3):e13297. doi: 10.1111/1758-2229.13297 (PMC11182661; doi:10.1111/1758-2229.13297)
Supplement: Supplementary file 1 — Supplemental Table 1. Microcystin concentrations (μg L−1) in 2022 of sampling sites for both cruises, Homa Bay drinking water plant intake, and Homa Bay pier. [file EMI4-16-e13297-s001.docx]

Supplemental Table 1. Microcystin concentrations (µg L^-1^) in 2022 of sampling sites for both cruises, Homa Bay drinking water plant intake, and Homa Bay pier.

| Site Name | Microcystins (µg L^-1^) | |
| --- | --- | --- |
|  | Cruise A | Cruise B |
| Kisumu Pier | < 0.15 | < 0.15 |
| Dunga | < 0.15 | < 0.15 |
| Nyando R. mouth | < 0.15 | < 0.15 |
| Sondu R. mouth | < 0.15 | < 0.15 |
| Kendu Bay | < 0.15 | < 0.15 |
| Bala Rawi | < 0.15 | < 0.15 |
| Gingra | < 0.15 | < 0.15 |
| Oluch | < 0.15 | 0.18 |
| Homa Bay | 0.19 | 2.22 |
| Soklo | < 0.15 | – |
| Mirunda Bay | 0.15 | 1.32 |
| Mbita East | < 0.15 | < 0.15 |
| Naya | < 0.15 | < 0.15 |
| Achieng Oneko | 0.15 | 0.18 |
| Asembo Bay | < 0.15 | ­– |
| Ndere Island | < 0.15 | 0.17 |
| Mid Gulf | < 0.15 | 0.16 |
| Maboko Island | < 0.15 | < 0.15 |
| Homa Bay Pier | 1.3 | – |
| Homa Bay Intake | < 0.15 | – |
