# Supplementary figures and images for: Bacterial community and cyanotoxin gene distribution of the Winam Gulf, Lake Victoria, Kenya
Source: Environ Microbiol Rep. 2024 Jun 17;16(3):e13297. doi: 10.1111/1758-2229.13297 (PMC11182661; doi:10.1111/1758-2229.13297)

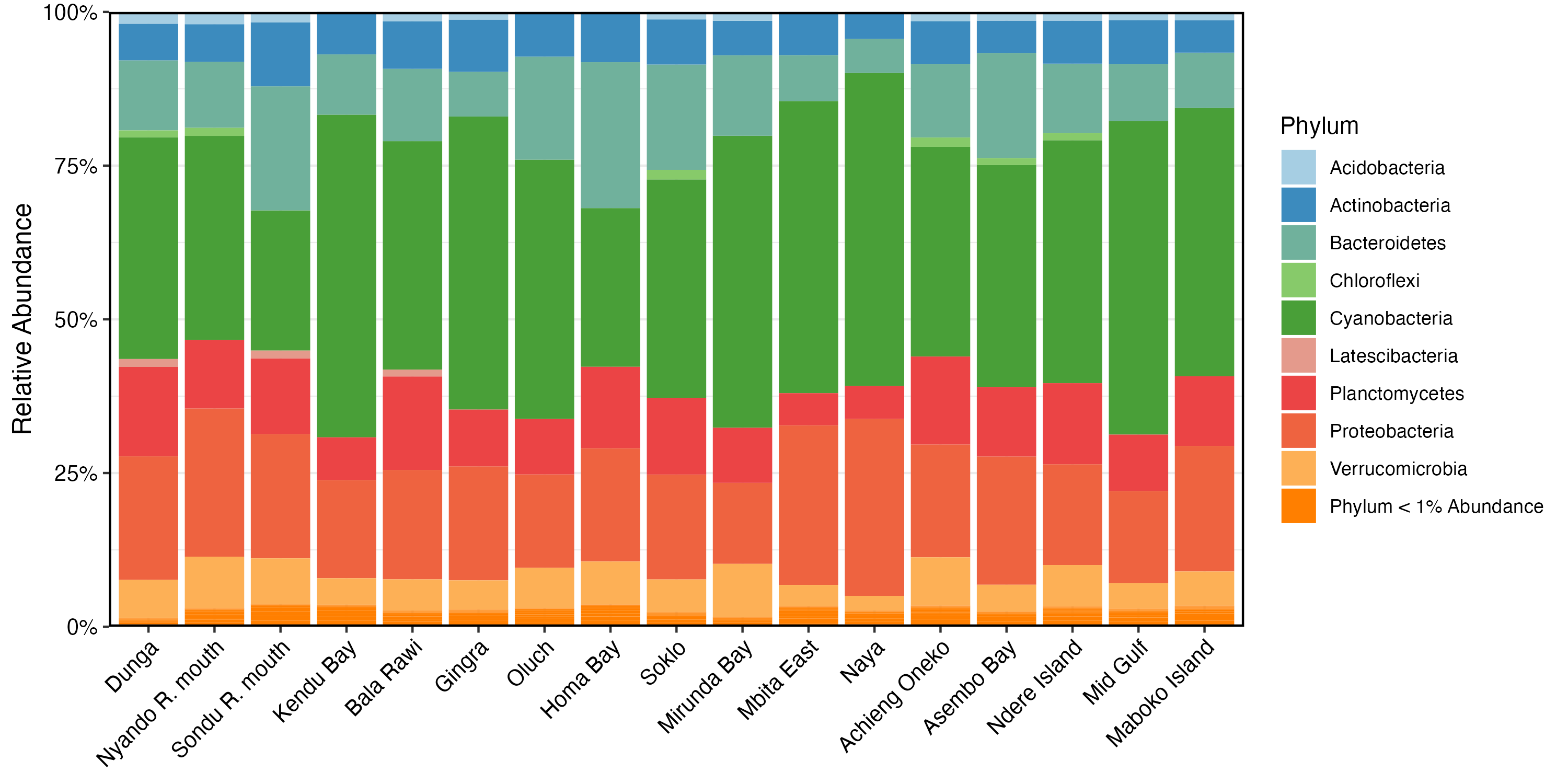

Supplement: Supplementary file 2 — Supplemental Figure 1. Relative abundance of prokaryotes at the phylum level. [file EMI4-16-e13297-s002.tiff]

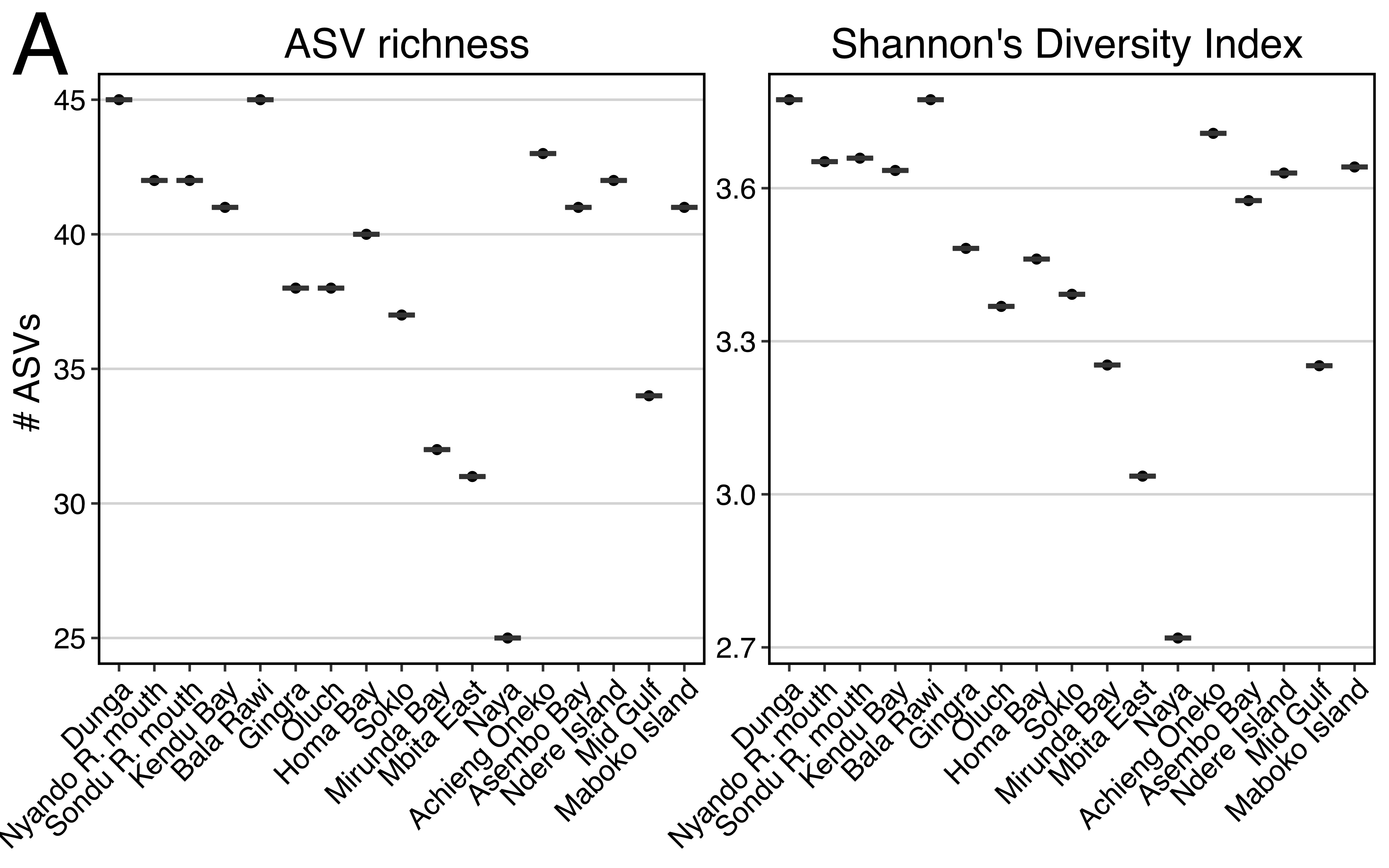

Supplement: Supplementary file 3 — Supplemental Figure 2. (A) Prokaryotic alpha diversity. (B) Cyanobacteria‐only alpha diversity. [file EMI4-16-e13297-s003.zip › S2a_All_AlphaDiv.tiff]

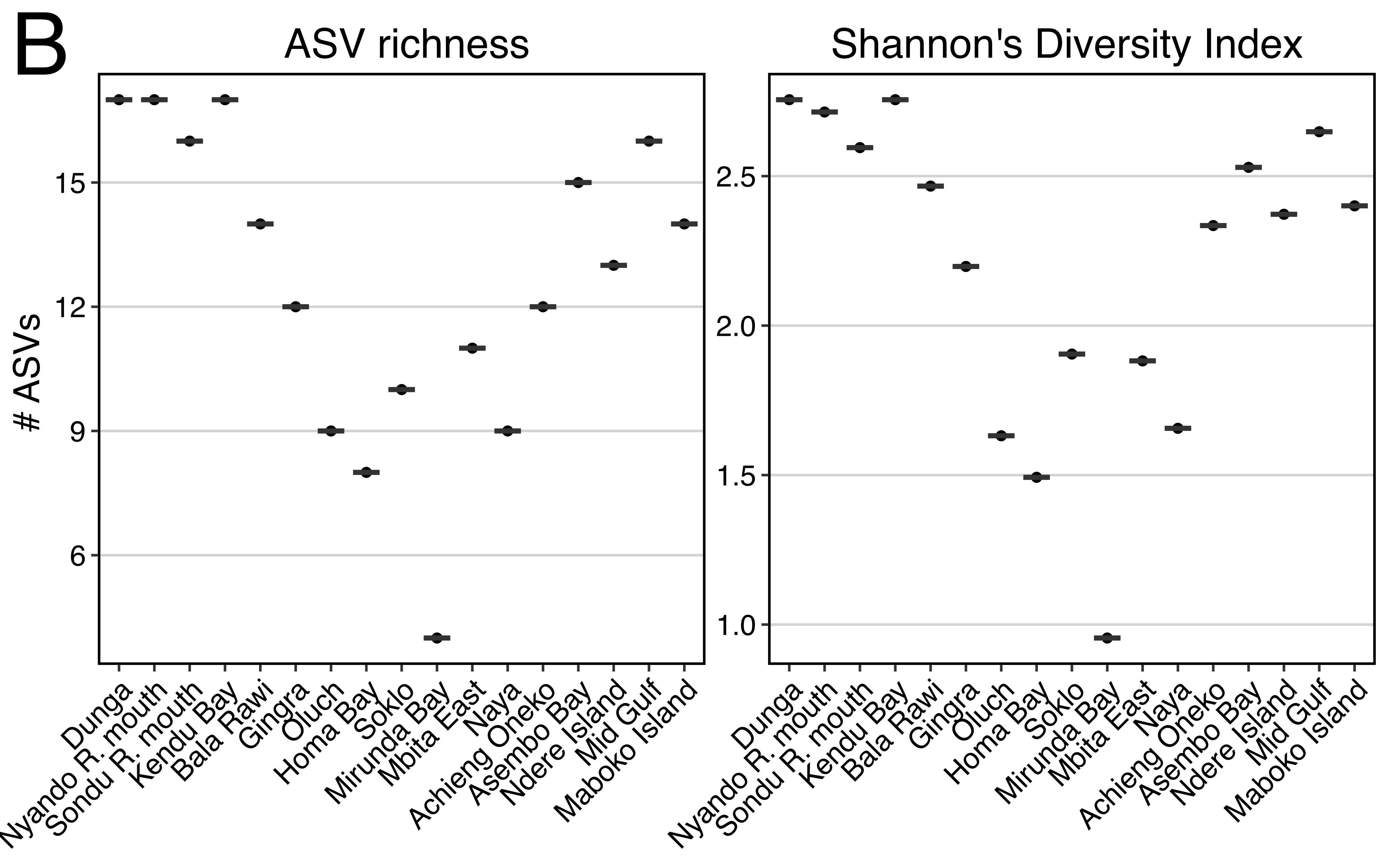

Supplement: Supplementary file 3 — Supplemental Figure 2. (A) Prokaryotic alpha diversity. (B) Cyanobacteria‐only alpha diversity. [file EMI4-16-e13297-s003.zip › S2b_Cyano_AlphaDiv.tiff]
